# Complications and safety aspects of kyphoplasty for osteoporotic vertebral fractures: a prospective follow-up study in 102 consecutive patients

**DOI:** 10.1186/1754-9493-2-2

**Published:** 2008-01-15

**Authors:** Yohan Robinson, Sven Kevin Tschöke, Philip F Stahel, Ralph Kayser, Christoph E Heyde

**Affiliations:** 1 Charité – Campus Benjamin Franklin, Centre for Trauma and Reconstructive Surgery, Berlin, Germany; 2 Denver Health Medical Center, Department of Orthopedic Surgery, University of Colorado School of Medicine, Denver, CO, USA

## Abstract

**Background:**

Kyphoplasty represents an established minimal-invasive method for correction and augmentation of osteoporotic vertebral fractures. Reliable data on perioperative and postoperative complications are lacking in the literature. The present study was designed to evaluate the incidence and patterns of perioperative complications in order to determine the safety of this procedure for patients undergoing kyphoplasty.

**Patients and Methods:**

We prospectively enrolled 102 consecutive patients (82 women and 20 men; mean age 69) with 135 operatively treated fractured vertebrae who underwent a kyphoplasty between January 2004 to June 2006. Clinical and radiological follow-up was performed for up 6 months after surgery.

**Results:**

Preoperative pain levels, as determined by the visual analogous scale (VAS) were 7.5 +/- 1.3. Postoperative pain levels were significantly reduced at day 1 after surgery (VAS 2.3 +/- 2.2) and at 6-month follow-up (VAS 1.4 +/- 0.9). Fresh vertebral fractures at adjacent levels were detected radiographically in 8 patients within 6 months. Two patients had a loss of reduction with subsequent sintering of the operated vertebrae and secondary spinal stenosis. Accidental cement extravasation was detected in 7 patients in the intraoperative radiographs. One patient developed a postoperative infected spondylitis at the operated level, which was treated by anterior corporectomy and 360 degrees fusion. Another patient developed a superficial wound infection which required surgical revision. Postoperative bleeding resulting in a subcutaneous haematoma evacuation was seen in one patient.

**Conclusion:**

The data from the present study imply that percutaneous kyphoplasty can be associated with severe intra- and postoperative complications. This minimal-invasive surgical procedure should therefore be performed exclusively by spine surgeons who have the capability of managing perioperative complications.

## Background

Osteoporotic vertebral compression fractures (VCF) are an epidemic burden disabling temporarily or permanently millions of elderly people worldwide. The annual incidence of VCF is 1.21% in women and 0.68% in men, increasing markedly with age [1]. With the continued aging of our population, VCF represent an important cause of disability and a significant source of healthcare resource utilization [2]. Non-surgical management with pain control and physical therapy-assisted mobilization is an effective treatment option. However, many patients remain immobilized due to chronic back pain [3]. The obvious functional and physical consequences of VCF lead to anxiety, depression, and have devastating impact on interpersonal relationships and social roles [4]. Strikingly, VCF have been shown to contribute significantly to shorter life-expectancy both in women (p < 0.01) and men (p < 0.0001) within one year after onset of symptoms [5].

Since restoration of quality of life has grown into a major issue in VCF treatment, operative treatment for pain reduction and correction of deformity has been much sought-after. Galibert et al [6] presented the first cases of successful vertebral augmentation by intravertebral injection of polymethyl methacrylate (PMMA) in patients with vertebral haemagiomas. Later, vertebroplasty was successfully introduced for the management of osteoporotic compression fractures [7]. The primary goal of vertebroplasty is pain relief by stabilization of the continuously sintering VCF [8]. A significant drawback of vertebroplasty is the fact that prevalent kyphosis cannot be corrected through this procedure. The biomechanical principle of increasing anterior column load with progressing kyphosis leading to subsequent VCF established the basic rationale for kyphoplasty. With this technique, reduction of VCF is achieved by a transpedicular intracorporal balloon expansion and retention by PMMA cement augmentation [9]. Up to the present, the concept of kyphoplasty has been successfully applied in thousands of patients with VCF, by decreasing fracture-related pain, improving biomechanics of the spine, as well as pulmonary function and quality of life [10,11].

Although kyphoplasty is performed in a minimally-invasive, percutaneous technique, the cement augmentation of vertebral fractures is associated with intra- and postoperative complications [12]. The most commonly described complication is an extravertebral leakage of PMMA cement through the venous system and through vertebral fracture cracks [13]. This can lead to spinal stenosis or to pulmonary cement embolism [14]. Even though kyphoplasty has significantly lower rates of cement extravasation than vertebroplasty [15], cement leakage may occur more frequently than appreciated and is associated with a significant morbidity [12]. In addition, systemic allergic or toxic reactions to PMMA monomers have been described [16]. Furthermore, the stabilization of a specific fracture level by kyphoplasty may lead to secondary fractures of adjacent vertebrae due to the changed biomechanics of the spine [17]. The presented study was designed to assess the overall incidence and patterns of complications related to the kyphoplasty procedure in patients with osteoporotic vertebral fractures.

## Patients and methods

This prospective study was performed between 1/2004 and 6/2006. Written informed consent was obtained from all participating individuals. A total number of 102 patients (82 women and 20 men; age: 69 ± 8 years) presented with 130 fractured vertebrae type A1 and 5 fractured vertebrae type A3 according to the AO classification by Magerl et al [18] (72 thoracic and 63 lumbar levels). The activity of osteoporotic VCF was confirmed in all cases by evaluation of the bony oedema in fat suppressed sequences (i.e. TIRM or STIR) of the magnetic resonance imaging (MRI). Furthermore anterior-posterior (a-p) and lateral radiographs were performed to evaluate the scoliotic and kyphotic deformity. Indication for kyphoplasty was kyphotic deformity >15 degrees, subsequent sintering (progressive loss of vertebral height), and pain resistant to analgesics and physical therapy for twelve weeks, as assessed by a visual analogue scale (VAS) score of more than 5 points.

As operative procedure we chose in 130 cases a percutaneus balloon kyphoplasty (Kyphon Inc, Sunnyvale, CA) with PMMA cement (Kyphx-HVR; Kyphon Inc.) after closed reduction through traction and lordosation under general anaesthesia. In the 5 cases with A3.1 fractures an additional stabilization with an internal fixator was performed (Universal Spine System; Synthes, Bettlach, Switzerland). During the procedure no biopsies were performed for pathologic review to rule out tumour as a cause of the pathologic bon quality. Peri- and postoperatively all patients received thrombosis prophylaxis with low-molecular weight heparins. Perioperatively all patients received an intravenous single-shot 1.5 g cefuroxime.

Follow-up was done 6 weeks, 12 weeks, and 6 months postoperatively. At follow-up plain radiographs were performed, and cement leakages, subsequent fractures, and further sintering (vertebral height and kyphosis) were determined.

## Results

Preoperative pain levels, as determined by VAS were 7.5 +/- 1.3. Postoperative pain levels were significantly reduced at day 1 after surgery (VAS 2.3 +/- 2.2) and at 6-month follow-up (VAS 1.4 +/- 0.9).

**Figure 1:**
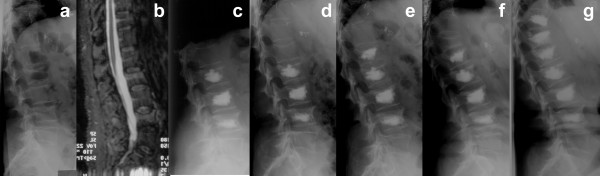
A 56-year old lady presented with painful compression fractures at L2, L3, and L4 due to corticoid-induced secondary osteoporosis **(a, b)**. As pain did not improve during non-surgical therapy for 6 weeks, kyphoplasty at L2–L4 was performed **(c)**. Two weeks postoperatively the patient reported again severe back pain. The radiographs revealed an adjacent compression fracture at L1 **(d)**. Therefore a kyphoplasty at L1 was performed **(e)**. One month later the patient presented again with severe thoracolumbar back pain, because of an adjacent fracture at T12 **(f)**. After kyphoplasty of T12 and prophylactic kyphoplasty of T11 the patient remained without further fractures **(g)**.

**Figure 2:**
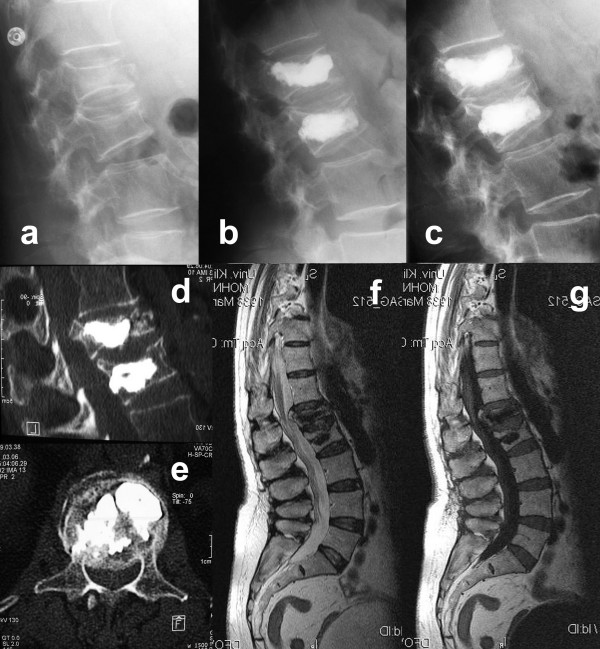
A 68-year old lady fell on glazed frost and presented with acute back pain without neurological symptoms. The plain radiographs revealed osteoporotic fractures at L1 and L2 type A1.2 according to Magerl et al [18] without spinal stenosis in both CT and MRI **(a)**. After kyphoplasty L1 and L2 and onset of a medical anti-osteoporotic therapy the patient was pain-free for one month **(b)**. The kyphotic deformity of L1 could be improved from 12 degrees to 6 degrees. Then she presented with immobilizing radicular pain radiating into the lumbar region. Signs of caudal or conus compression were not present. Plain radiographs revealed a sintering of the already kyphoplastized vertebra L1 with 14 degrees kyphosis **(c)**. CT-scans revealed a significant central and foraminal stenosis **(d, e) **without myelon compression in the MRI **(f, g)**. After a microscopically-assisted decompression at T12/L1 the patient was pain-free and further sintering did not occur thereafter.

**Figure 3:**
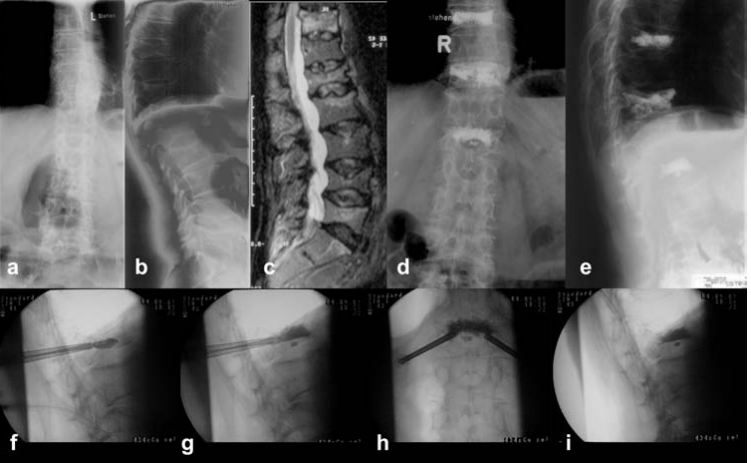
A 77-year old man complained about severe thoracolumbar back pain. Plain radiographs revealed multiple osteoporotic vertebral compression fractures **(a, b)**, of which fractures at Th9, Th11, and L1 were relatively fresh in the stir-sequence of the MRI **(c)**. Because of severe pain resistant to non-surgical therapy for 2 months we decided to perform kyphoplasty at Th9, Th11, and L1. During the procedure the flattened vertebra L1 was impossible to reduce **(f)**, while filling the vertebra with PMMA cement a leakage occurred into the lower disc **(g-i)**. Nevertheless the patient had dramatically reduced back pain, presented no sign of neurological damage and was released two days after the procedure **(d, e)**.

**Figure 4:**
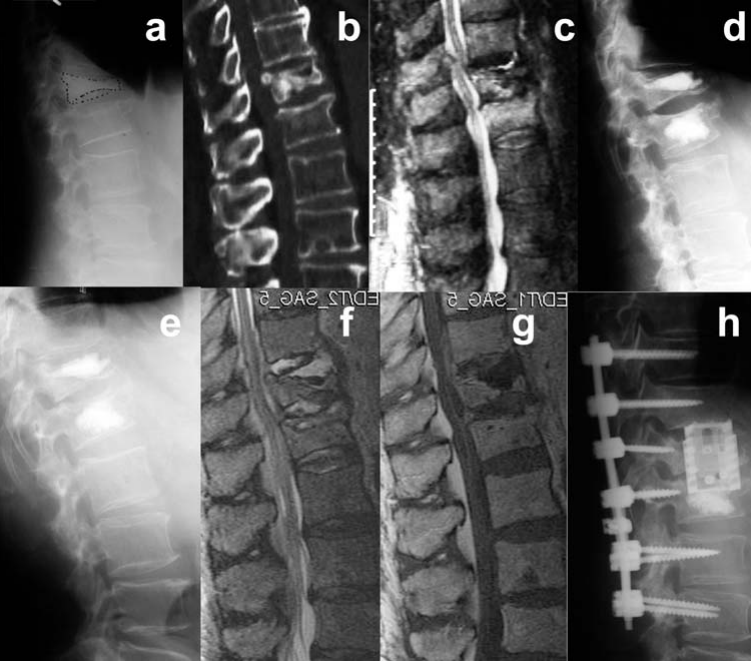
This 68-year old man with corticoid-induced secondary osteoporosis and multiple co-morbidity fell at home and presented with osteoporotic fractures at T12 and L1 **(a, b)**. The MRI confirmed fresh fractures and revealed a spinal stenosis at T12/L1 **(c)**. Since non-surgical therapy was not successful, neurological deficits were not prevalent, kyphoplasty at T12 and L1 was performed as a minimal intervention **(d)**. Postoperatively the patient was mobilised and left the hospital 4 days after kyphoplasty. Two weeks later the patient was admitted to our emergency care unit with incomplete paraplegia sub T8. Laboratory diagostics revealed highly elevated leukocytes and C-reactive protein. Plain radiographs showed a thin radiolucency around the cement core on T12 **(e)**. The MRI confirmed the suspected spondylitis and found additionally an epidural abscess **(f, g)**. Therefore posterior decompression with instrumentation from T10 to L3 was performed and anterior corporectomy of T12 with complete cement removal and implantation of an expandable titanium-cage and bone graft was performed **(h)**. An incomplete paraplegia sub L2 remained.

This study focused on complications of kyphoplasty, which could be distinguished between late complications (subsequent VCF, secondary stenosis through sintering) and early/perioperatively (cement leakage, infection, haematoma). Complications occurred in 18 patients (17.6%) Fresh vertebral fractures at adjacent levels were detected radiographically in 8 patients (7.8%) within 6 months (Fig. 1). Two patients (2.0%) had a loss of reduction with subsequent sintering of the operated vertebrae and secondary spinal stenosis (Fig. 2). Accidental cement extravasation was detected in 7 patients (6.9%) in the intraoperative radiographs (Fig. 3). One of these leakages (1.0%) was into the spinal canal without any relevant spinal stenosis or neurological compromise. No sign of cement embolisms or allergic reactions to PMMA monomers was seen. One patient (1.0%) developed two weeks after kyphoplasty a postoperative infected spondylitis at the operated level with epidural abscess and incomplete paraplegia, which was treated emergently by posterior decompression, abscess evacuation, and instrumentation as well as anterior debridement and corporectomy in a 360 degrees fusion (Fig. 4). Another patient (1.0%) developed a superficial wound infection after 10 days which required surgical revision. Postoperative bleeding resulting in a subcutaneous haematoma evacuation was seen in one patient (1.0%).

## Discussion

Fourteen years after the first vertebroplasty was performed in 1984, the procedure met its worst competitor promising less complications and reconstructive ability: kyphoplasty. Until now, several non-randomized prospective controlled trials have been published comparing kyphoplasty to non-surgical treatment and vertebroplasty (Table 1) and four ongoing randomized controlled trials are registered (Table 2) [19]. Major issues are pain improvement and quality of life, correction of deformity and postoperative complications. The first results of the multicentrical randomized controlled Fracture Reduction Evaluation (FREE) study present a significant improvement of the quality of life (SF-36, p < 0. 01) after 3 months in the kyphoplasty group (n = 149) controlled against non-surgical treatment (n = 151) [11]. Only one device-related serious adverse effect (a soft tissue haematoma) has been reported in the FREE study, but first the 1-year results, which will be published soon, will give evidence concerning the safety of kyphoplasty.

**Table 1:** Overview on comparative clinical trials (CT) of kyphoplasty

**Author**	**Year**	**Design**	Level of evidence*	**Control Group**	**Control n (levels)**	**Kyphoplasty n (levels)**	**Follow-up**	**Outcome**	**Cement leakage**
Weisskopf et al. [56]	2003	Retrospective CT	IIb	non-surgical	20 (35)	22 (37)	10 days	Improvement in VAS (p < 0.001) Reduced days in hospital (p < 0.01)	5 cement leakages in kyphoplasty
Fourney et al. [57]	2003	Retrrospective CT	IIb	vertebroplasty	34 (65)	15 (32)	4,5 months	No significant differences in VAS and ODI Improvement of kyphosis with kyphoplasty (p < 0.01)	0 cement leakages in kyphoplasty 6 cement leakages in vertebroplasty
Komp et al. [58]	2004	Prospective CT	IIa	non-surgical	19(19)	21(21)	6 months	Improvement of VAS and ODI (p < 0.01)	0 cement leakages in kyphoplasty
Kasperk et al [59]	2005	Prospective CT	IIa	non-surgical	20 (33)	40 (72)	12 months	Improvement of VAS (p < 0.01) Improve of kyphosis (p < 0.001).	7 cement leakages in kyphoplasty
Grohs et al. [60]	2005	Prospective CT	IIa	vertebroplasty	23 (29)	28 (35)	24 months	No significant difference in ODI Improvement of VAS with kyphoplasty (p < 0.05) No significant improve of kyphosis	8 cement leakages in kyphoplasty 8 cement leakages in vertebroplasty
Masala et al. [61]	2005	Retrospective CT	IIb	vertebroplasty	26 (33)	7 (7)	6 months	No significant difference in VAS.	0 cement leakage in kyphoplasty 11 cement leakages in vertebroplasty
Pflugmacher et al [62]	2005	Prospective CT	IIa	vertebroplasty	20 (32)	22 (35)	12 months	No significant difference in VAS and ODI Improvement of kyphosis with kyphoplasty (p < 0.05)	5 cement leakages in kyphoplasty 6 cement leakages in vertebroplasty
De Negri et al. [63]	2007	Prospective CT	IIa	vertebroplasty	10 (18)	11 (15)	6 months	No significant difference in VAS and ODI	0 cement leakages in kyphoplasty 1 cement leakage in vertebroplasty
Frankel et al. [64]	2007	Retrospective CT	IIb	vertebroplasty	19 (26)	17 (20)	6 months	No significant difference in VAS Higher rate of adjacent fractures with kyphoplasty (p < 0.05)	3 cement leakages in kyphoplasty 2 cement leakages in vertebroplasty
Müller et al [11]	2007	Randomized CT	Ib	non-surgical	149	151	3 months	Improvement in SF-36 (p < 0.01) and VAS (p < 0.01) with kyphoplasty	Not reported

* Levels of evidence according to the recommendations of the US Agency for Health Care Policy and Research

VAS: Visual Analogous Scale, ODI: Oswestry Disability Index, SF-36: MOS-36 Item Short Form Health Survery

**Table 2:** Registered ongoing multicenter randomized controlled trials involving kyphoplasty [19]

**Trial name**	**Procedure**	**Control group**	**n**	**Follow-up**	**Primary outcome**
FREE	Kyphoplasty in VCF	non-surgical	300	2 years	Quality of life (SF-36)
CAFE	Kyphoplasty in VCF in cancer patients	non-surgical	200	1 year	Pain (VAS), Disability (Roland-Morris)
CEEP	Kyphoplasty in VCF	vertebroplasty	112	2 years	Pain (Roland-Scale)
KAVIAR	Kyphoplasty in VCF	vertebroplasty	1,234	2 years	Subsequent fractures

VCF: Vertebral compression fracture, VAS: Visual Analogous Scale, SF-36: MOS-36 Item Short Form Health Survey

### Correction of deformity

Osteoporotic VCF lead to significantly reduced life expectancy in both men and women [5]. In postmenopausal women the risk of subsequent VCF is much greater than for other fractures (relative risk = 4.4) [20,21]. This risk increases with the severity of the deformity [22]. Therefore surgical correction of deformity has the ability to reduce morbidity and mortality in these patients. Conventional open interventions require anterior open-wedge or posterior closing-wedge techniques with long distance posterior fusions because of poor bone quality. Due to the surgical access and co-morbidities these operations go along with severe complications, but they were found to have excellent 2-year results in ODI and VAS improvement [23]. Much less invasive is the kyphoplasty procedure, which also has the capability of reconstructing VCF height. Voggenreiter et al [9] found a reduction of 3.1 degrees Cobb angle in standing radiographs after kyphoplasty (n = 30). Pradhan et al [24] found a local correction of the fractured vertebra of 7.2 degrees, but only 2.4 degrees of the Cobb angle, when measured 1 level above and below (n = 65). Interestingly they found a greater improvement of kyphosis with multilevel kyphoplasty of 7.7 degrees Cobb angle.

### Complications after Kyphoplasty

The comprehensive meta-analysis of Taylor et al [19] summarized all published kyphoplasty complications. Cement leakages occurred in 8.1% of all cases, but only 0.09% were symptomatic. New vertebral fractures occurred in 11.1%, and 9.4% were adjacent vertebrae. Pulmonary embolism occurred in 0.17% of all cases. Spinal stenosis with spinal cord compression occurred in 0.16% of all cases. Radiculopathy was found in 0.17% of all cases. The overall mortality was 4.4%, perioperative mortality was 0.13%.

### Adjacent vertebral fractures

Adjacent fractures are the most common adverse event found after kyphoplasty. The occurrence of adjacent fractures is known from vertebroplasty, where 12.4% had subsequent VCF after two years (n = 177) [25]. Kyphoplasty was thought to have lesser adjacent fractures due to correction of kyphotic deformity [22], but kyphosis is not the only reason for adjacent fractures. Lin et al [26] correlated the incidence of adjacent fractures to cement leakage into the disc in vertebroplasty (n = 38, p < 0.005). Komemushi et al [27] found cement leakage into the disc to be a significant predictor of adjacent VCF (n = 83, p < 0.001). These findings will apply to cement leakage in kyphoplasty, too.

In our study population after 6 months adjacent fractures were found in 7.8% of all cases (n = 102). Fifty percent of these patients had secondary osteoporosis due to corticoid medication. Fribourg et al [17] found subsequent fractures after kyphoplasty in 26% of all cases (n = 38), 21% occurred during the first two months. Harrop et al [28] found subsequent fractures in 22.6% of all patients after a mean follow-up of 11 months (n = 115). Sixty-five percent of these had secondary steroid-induced osteoporosis. Another investigation by Lavelle & Cheney [29] found 17% recurrent fractures within one year after kyphoplasty, 11.7% occurred during the first 90 days (n = 94). They did not find any impact of secondary osteoporosis on subsequent fractures. A prospective investigation by Moon et al [30] found an incidence of subsequent VCF in 15.5% patients after one year (n = 111). Interestingly they could correlate the appearance of adjacent VCF to the amount of PMMA cement applied during the procedure (p < 0.05).

The available data reveals that subsequent VCF of adjacent vertebrae occur in 7.8% to 26% of all patients treated with kyphoplasty. Kyphosis, secondary osteoporosis, and cement leakage into the intervertebral disc facilitate the occurrence of subsequent VCF. Identifying risk factors for subsequent VCF, several authors discussed the indication for prophylactic cement augmentation of adjacent vertebrae [[31-33]]. Especially in cases of "sandwich-kyphoplasty" with an osteoporotic non-fractured vertebra between two kyphoplasties some recommend a prophylactic kyphoplasty [32]. Until now there is no clinical evidence for the effectiveness of prophylactic vertebro- or kyphoplasty [32], but the biomechanical investigation of Sun and Liebschner [31] using finite-elements found a significant reinforcement of high-risk vertebral bodies with prophylactic vertebroplasty. Despite some promising experimental data, in the light of the present evidence it rather seems that the prophylactic use of PMMA reduces the safety of the procedure [31].

Fearing secondary spinal stenosis due to further sintering of burst fractures after kyphoplasty several surgeons perform posterior instrumentation of the adjacent vertebrae to protect the posterior wall [34]. This can be done using percutaneous posterior instrumentation or with a conventional open technique. Verlaan et al [35] investigated the use of kyphoplasty after posterior instrumentation in burst fractures in 20 patients (mean age 41.8 years). No bone fragment displacement was found. Unsymptomatic cement leakage occurred in 5 cases. Vertebral anterior height could be restored to 91% of the estimated intact height. Nöldge et al [34] performed kyphoplasty with posterior instrumentation in 9 patients with burst fractures. They found a reduction of mean VAS of 6.2 preoperatively to 2.0 after one year. Unfortunately the evidence supporting the additional instrumentation after kyphoplasty is very low. Therefore this procedure is promising, but it has to be evaluated prospectively in the future.

### Cement leakage

A feared complication of all vertebral augmentation techniques is PMMA cement leakage. The systematic review of the literature by Hulme et al [12] found rates of cement leakage in vertebroplasty of 41% (n = 2,283 levels) and in kyphoplasty of 9% (n = 1,486 levels) of treated vertebrae.

In the presented study we found radiographically confirmed cement leakage in 6.9% of all cases. These results are lesser than average, but cement leakage was only identified on plain radiographs. CT-scans identify more leaks than radiographs by a factor of 1.5 [36]. In kyphoplasty of the 65 leakages reported in the literature most were paraspinal (48%), intradiscal (38%), epidural (11%), pulmorary (1.5%) and foraminal (1.5%) [12]. Paraspinal and intradiscal leakages generally are asymptomatic, even though intradiscal leakage is blamed to promote adjacent fractures [27]. Intradural cement leakage has only been described for vertebroplasty so far [37], but epidural leakage had devastating neurological effects both in vertebroplasty [38] and in kyphoplasty [39]. These complications can require immediate surgical intervention with decompression and, if possible, removal of the cement causing stenosis [40].

Pulmonary embolism of PMMA cement was found in 4.6% of the 65 patients treated with either vertebroplasty (n = 88) or kyphoplasty (n = 25) by Choe et al [14]. No correlation between the occurrence of pulmonary cement embolism and the type of procedure was found. This is remarkable since kyphoplasty has a much lesser rate of cement leakage than vertebroplasty [12]. Pulmonary cement embolism rarely requires intervention and mostly remains asymptomatic. Often they are accidental findings in chest radiographs, but there are several case reports with clinically relevant cement embolisms. Jang et al [41] presented three cases of cement embolisms after vertebroplasty, of which two had mild dyspnoea. No pulmonary perfusion defects were seen and intervention was not necessary. More severe is the case of François et al [42] with a large PMMA cement embolus floating in the right pulmonary artery menacing pulmonary function. Therefore the embolus was removed by open heart surgery. In a case report by Yoo et al [43] a 5 cm long PMMA cement embolus in the right pulmonary artery after vertebroplasty lead to acute respiratory distress syndrome, requiring intensive care treatment and open embolectomy under cardiopulmonary bypass. The patient did not recover and died ten days after vertebroplasty. A further fatal pulmonary embolism after vertebroplasty has been described by Monticelli et al [44]. There is only one report on cement embolism after kyphoplasty by Garfin et al. [45]. Reports on lethal pulmonary cement embolism after kyphoplasty do not exist.

With proper surgical techniques the risk of cement leakage can be minimized. Correct placement of the balloon, high viscosity of the PMMA cement, controlled application of the cement in to the vertebra, and limitation of the applied volume reduce the risk of leakage. A popular technique to reduce the risk of cement leakage in kyphoplasty is the eggshell-technique, where after primary reduction with the balloon a small amount of doughy cement is applied into the cavity followed by re-inflation of the balloon [46]. Using this technique a cement "eggshell" prevents further leakage when the rest of the cement is applied with radiographic control.

The severity of pulmonary PMMA cement embolism and the urgent need of immediate decompression in relevant spinal stenosis after cement leakage, questions the common practice of vertebroplasty and kyphoplasty in an outpatient practice, without any spinal surgeon on call, without any available operating theatres, and without an intensive care unit. Even though fatal embolisms are few kyphoplasty should not be regarded as minor intervention which can be performed without the availability of the above mentioned conditions and requirements.

### Infections

Only two cases of infections after kyphoplasty have been described so far in the available literature. Nussbaum et al [47] found two cases of infection (discitis/osteomyelitis) in the large "Food and Drug Administration" (FDA) database for adverse events related to kyphoplasty devices. The two presented cases with infection after kyphoplasty in this study reflect the risk of infection being adherent to every surgical intervention [48]. To our knowledge with vertebroplasty only seven cases have been described with postoperative infections. The vertebroplasty pioneers Deramond et al [7] presented one case of postoperative spondylitis in an immunosuppressed patient, which could be treated successfully by bedrest and antibiotics. Kallmes et al [49] described a case of a postoperative infection in an immunocompromised patient. Another case was presented by Yu et al [50] with severe pyogenic spondylitis one month after vertebroplasty which was performed while the patient had urinary tract infection. The treatment was surgical with anterior corporectomy and bisegmental fusion after multisegmental posterior instrumentation. A further case of spondylitis after vertebroplasty was presented by Schmid et al [51], which treated the patient conservatively with a 3-month antibiotic regimen. Walker et al [52] and Mummameni et al [53] present two additional cases of spondylitis after vertebroplasty treated by anterior corporectomy and multisegemental fusion. Alfonso Olmos et al [54] report a case of spondylitis after vertebroplasty requiring corporectomy and 360 degrees fusion. An unusual case report of spondylitis after vertebroplasty with epidural abscesses containing mycobacterium tuberculosis was published by Bouvresse et al [55]. This patient was under immunosuppression because of a liver transplantation and an inactive tuberculous lesion was obviously activated. Successful treatment implied posterior decompression, abscess evacuation, and long-term antituberculotic therapy.

Even though the risk of infection after kyphoplasty is extremely low, infection does occur, as we have demonstrated in this investigation. The available clinical experience from infection after vertebroplasty stresses the safety limits of vertebral cement augmentation in immunocompromised patients. In these cases a standardized antibiotic prophylaxis is recommended. Nevertheless the rate of postoperative infections is lower both in kyphoplasty and in vertebroplasty than in any other spinal surgical procedure in general [48].

## Conclusion

During the past five years kyphoplasty entered standard VCF treatment protocols, replacing vertebroplasty in many areas. Nevertheless conservative medical therapy will not be easily replaced, since lack of reimbursement in most countries causes an economic burden, many patients are not willing to take. Furthermore it is still unclear whether the benefits of kyphoplasty outweigh its complications. The results of the case series presented here demonstrate that kyphoplasty can be considered a safe procedure, if performed in a hospital-based setting. Although the overall complication rate is 15%, major complications are rare. However, since severe acute complications requiring emergency treatment may occur, we believe that the procedure should be performed by a qualified spine surgeon in a trauma center, exclusively.

## Competing interests

YR and CEH are Clinical Investigators of the "Fracture Reduction Evaluation" (FREE) trial, which is carried out and supported financially by Kyphon Inc., Sunnyvale, CA.

## Authors' contributions

YR carried out the study design, performed the data analysis, participated in the sequence alignment and drafted the manuscript. SKT, PFS, and RK participated in the sequence alignment and revision of the manuscript. CEH conceived of the study, and participated in its design and coordination and helped to draft the manuscript. All authors read and approved the final manuscript.
